# Resting-State Functional Magnetic Resonance Imaging for Surgical Neuro-Oncology Planning: Towards a Standardization in Clinical Settings

**DOI:** 10.3390/brainsci11121613

**Published:** 2021-12-07

**Authors:** Gianvincenzo Sparacia, Giuseppe Parla, Giuseppe Mamone, Mariangela Caruso, Fabio Torregrossa, Giovanni Grasso

**Affiliations:** 1 Radiology Service, Department of Biomedicine, Neurosciences and Advanced Diagnostics (BiND), University of Palermo, 90100 Palermo, Italy; gsparacia@ismett.edu; 2 Radiology Service, Department of Diagnostic and Therapeutic Services, Mediterranean Institute for Transplantation and Advanced Specialized Therapies (IRCCS-ISMETT), 90100 Palermo, Italy; gparla@gmail.com (G.P.); gmaimone@gmail.com (G.M.); 3 Neurosurgical Unit, Department of Biomedicine, Neurosciences and Advanced Diagnostics (BiND), University of Palermo, 90100 Palermo, Italy; caruso_mariangela@libero.it (M.C.); fabiotorregrossa00@gmail.com (F.T.)

**Keywords:** brain mapping, brain tumors, functional connectivity, resting-state fMRI

## Abstract

Resting-state functional magnetic resonance imaging (rest-f-MRI) is a neuroimaging technique that has demonstrated its potential in providing new insights into brain physiology. rest-f-MRI can provide useful information in pre-surgical mapping aimed to balancing long-term survival by maximizing the extent of resection of brain neoplasms, while preserving the patient’s functional connectivity. Rest-fMRI may replace or can be complementary to task-driven fMRI (t-fMRI), particularly in patients unable to cooperate with the task paradigm, such as children or sedated, paretic, aphasic patients. Although rest-fMRI is still under standardization, this technique has been demonstrated to be feasible and valuable in the routine clinical setting for neurosurgical planning, along with intraoperative electrocortical mapping. In the literature, there is growing evidence that rest-fMRI can provide valuable information for the depiction of glioma-related functional brain network impairment. Accordingly, rest-fMRI could allow a tailored glioma surgery improving the surgeon’s ability to increase the extent of resection (EOR), and simultaneously minimize the risk of damage of eloquent brain structures and neuronal networks responsible for the integrity of executive functions. In this article, we present a review of the literature and illustrate the feasibility of rest-fMRI in the clinical setting for presurgical mapping of eloquent networks in patients affected by brain tumors, before and after tumor resection.

## 1. Introduction

In the setting of brain tumors, the primary goal of the surgery aims to maximize the tumor resection while preserving brain function [1,2,3]. Resting-state functional magnetic resonance imaging (rest-fMRI) represents an emerging technique to assess brain functional network connectivity y providing useful information in the presurgical planning and the postoperative follow-up for brain tumor surgery [4]. Indeed, in the literature, there is evidence that rest-fMRI can demonstrate glioma-related functional brain network impairment [5,6]. In this scenario, high-grade gliomas (HGG) are characterized by biological properties which lead to rapid clinical progression and poor prognosis [7]. Maximal safe resection is one of the main prognostic factors to increase the survival of affected patients [8,9]. Moreover, current investigations have focused on pathophysiology mechanisms and therapeutic approaches to improve HHG patients’ outcome [10,11,12].

Rest-fMRI is a novel neuroimaging technique that explores functional connectivity networks associated with both normal and pathologic neurologic function [13]. It works at “rest,” which requires minimal participant collaboration, and may replace or can be complementary to task-driven fMRI, particularly in patients who are sedated, aphasic, or paretic, thus unable to cooperate for the acquisition of task-driven fMRI, or in children [13].

Although rest-fMRI is still under standardization, there is evidence that it can provide useful information about brain neuronal networks organization in the routine clinical setting. In particular, it can be used to in vivo map eloquent areas. Thus, rest-fMRI could be used for detecting glioma-related functional brain network alterations, improving the surgeon’s ability to increase the tumor resection and simultaneously minimize the risk of damage to brain neuronal networks responsible for executive functions integrity [5,6].

In this review, we illustrate the principles and the value of rest-fMRI in the clinical setting for presurgical mapping of neuronal networks in patients affected by brain tumors, and after tumor resection.

## 2. Principles of Rest-fMRI

**Figure 1:**
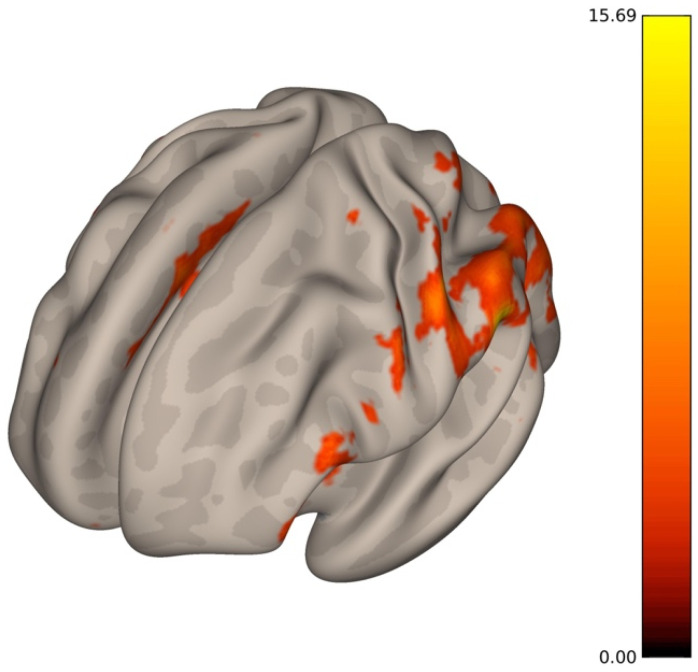
3D brain surface with color map overlay reflecting the spontaneous low-frequency BOLD signal fluctuation which implies activation of distinct patterns of cerebral areas during the resting state underlying the cerebral connectivity.

Rest-fMRI is an emerging neuroimaging technique depending on the quantification of hemodynamic changes following the activation of brain areas. The increase in neuronal activity involves a greater demand of energy, and therefore oxygen, by neurons. Thus, neuronal activity induces a hemodynamic response that alters local brain concentrations of oxyhemoglobin and deoxyhemoglobin. This process produces time-dependent alterations in T2- and T2*-relaxation times, constituting the basic principles of the blood oxygen level-dependent (BOLD) contrast imaging [13]. Since the oxyhemoglobin has a diamagnetic effect and the deoxyhemoglobin has a paramagnetic effect, voxels having a low concentration of deoxyhemoglobin increases the BOLD signal, and those with a high concentration contribute to a decrease in the BOLD signal. Different from task-driven fMRI, the rest-fMRI does not require any stimulus (i.e., motor, language, visual task). The result is a color brain map reflecting the spontaneous low-frequency BOLD signal fluctuation which implies activation of distinct patterns of cerebral areas during the resting state underlying the cerebral connectivity (Figure 1).

**Figure 2:**
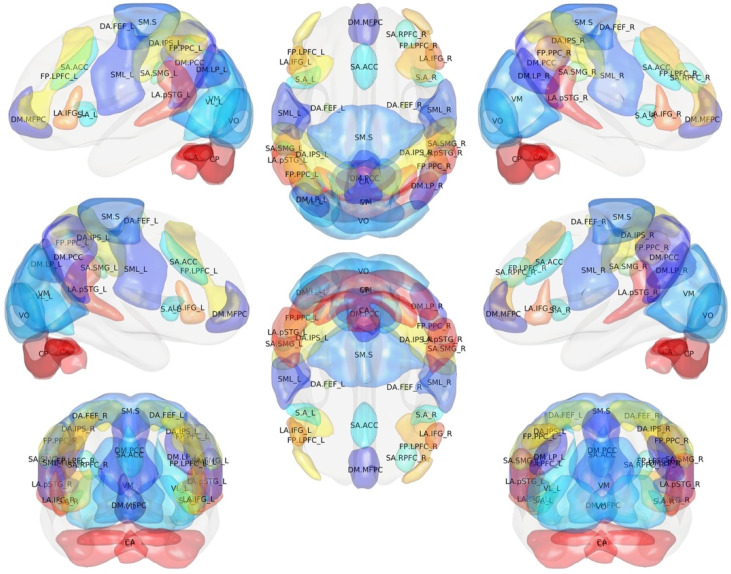
3D color brain map depicting resting-state functional magnetic resonance imaging (fMRI) functional regions. In each region, resting-state networks (RSNs) are subdivided on the basis of their different functions, such as the default mode network (DMN), the sensorimotor network, the auditory network, the visual network, the executive control network, the lateralized frontoparietal network, and the temporoparietal network.

The human brain networks are active even during the resting or relaxing state. Furthermore, resting-state brain activity is far more significant than task-related activity, consuming 60–80% of the brain’s energy [14]. These networks of the brain are called resting-state networks (RSNs) [6,15]. Rest-fMRI can reliably depict temporally coherent networks, and they are codified in the literature based on their different functions, such as the default mode network (DMN), the sensorimotor network, the auditory network, the visual network, the executive control network, the lateralized frontoparietal network, and the temporoparietal network [5,16,17,18,19] (Figure 2, Table 1).

**Figure 3:**
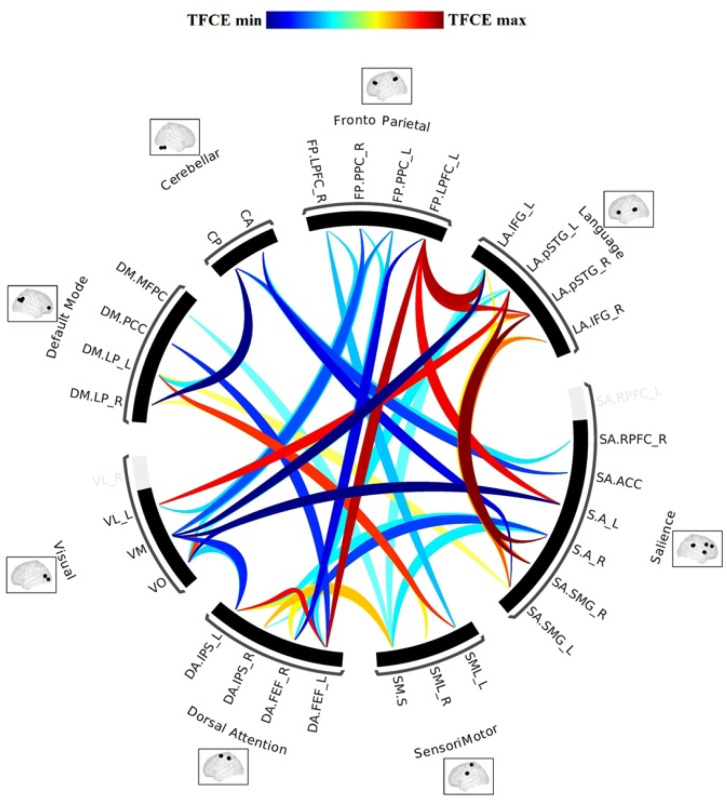
Graph representing the intrinsic functional architecture of the human brain RSNs named connectome.

The proof of the existence of the RSNs is based upon the reproducibility of the networks in the single subject, the consistency of the networks between different subjects, and the correspondence of the cortical areas identified with different methods of study [5,6,20,21]. Therefore, rest-fMRI is a valid method to evaluate the intrinsic functional architecture, or “connectome,” of the human brain [5] (Figure 3) with high reproducibility and reliability.

Brain MR imaging accurately demonstrates the morphological aspects of brain tumors with standard sequences, such as the three-dimensional (3D) magnetization-prepared rapid acquisition with gradient echo (MPRAGE) T1-weighted images obtained before and after gadolinium enhancement. Echo-planar imaging (EPI) sequence for rest-fMRI can be performed in a routine clinical setting, instructing the patient to stay still with their eyes closed while relaxing [5,6]. The rest-fMRI sequence is largely available on MRI scanners. By contrast, the main limitation for the clinical implementation of rest-fMRI is the lack of methodology standardization for data processing, which is based on a variety of different software tools and methods that require trained personnel and high skill of expertise [5,6].

## 3. Clinical Application of Rest-fMRI

Rest-fMRI is an emerging technique valuable for the exploration of brain physiology. Recent studies have explored the potential applications of rest-fMRI in various neurologic diseases (e.g., epilepsy, and neurodegenerative and psychiatric diseases) [22,23,24,25]. However, the value of rest-fMRI in the neurosurgical planning for brain tumors was not fully investigated [6,15,26,27,28,29,30,31].

Several studies reported high overlap between rest-fMRI and task-driven fMRI in various neurologic diseases when comparing the motor network, as well as high concordance with cortical stimulation mapping [32]. Other studies have demonstrated high reproducibility of rest-fMRI-derived motor maps comparable to that of task-driven fMRI in healthy subjects [33].

**Figure 4:**
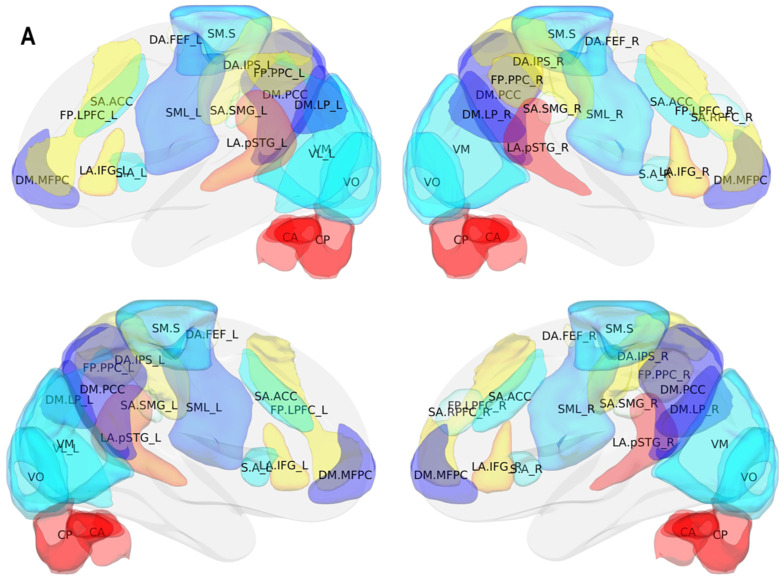

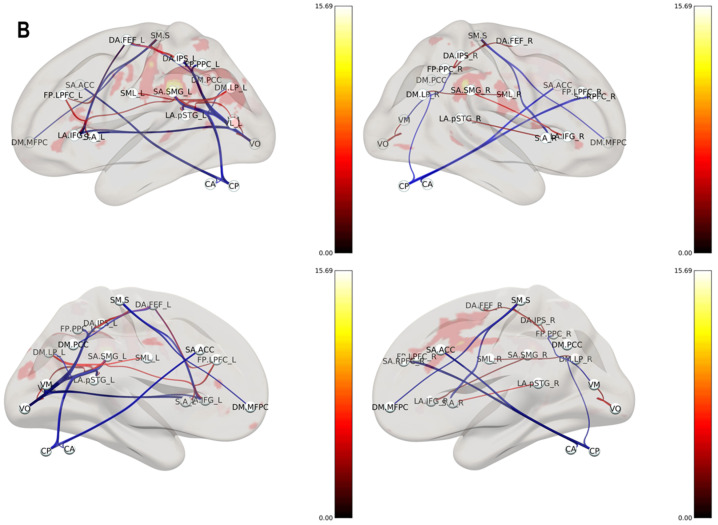
3D color brain maps showing several functional RSNs, such as the language, visual, and sensorimotor network, with precise cortical parcellation identification. Different RSNs depict specific functions and their spatial location. 3D color brain maps showing (**A**) several functional RSNs, such as the language, visual, and sensorimotor network, with precise cortical parcellation identification. Different RSNs depict specific functions and (**B**) their functional connection.

Resting-state fMRI can reliably detect common functional connectivity networks in patients with glioma, and has the potential to anticipate network alterations after surgical resection. Alterations of RSNs can be analyzed at the level of the single subject and in group analysis, with a functional mapping demonstrating a good correspondence with cortical stimulation mapping [32]. Although task-driven-fMRI is used to provide preoperative localization of eloquent areas to reduce the risk of surgery-induced functional deficits, several functional networks can be identified using rest-fMRI, such as language, visual, and sensorimotor networks with precise cortical parcellation identification [34,35] (Figure 4). This MR technique does not require any patient activity and can be performed in young children and in patients who are unable to collaborate due to being aphasic, paretic, or sedated patients. Functional connectivity in patients with brain tumors can be carried out by recognizing the different RSNs and their alterations [36,37,38,39]. Different RSNs can be evaluated with the same sequence and in the same examination, showing normal connectivity or impairment of selected functional RSNs.

Clinical presurgical rest-fMRI can be merged with standard anatomic sequences (FLAIR, T1-precontrast, and postcontrast) and imported onto the neuronavigational system, thus can be used to guide intraoperative stimulation.

## 4. Postprocessing of Resting-State fMRI and Analysis of Brain Networks

Rest-fMRI evaluation of functional brain connectivity has demonstrated different RSNs, which depict specific functions and varied spatial location [6,15,18] (Figure 4). Anatomical connectivity is the physical connections for the interactions between two anatomical areas of the brain, whereas functional connectivity represents the connection between two or more spatial regions whose activity shows a linear temporal correlation.

Different post-processing and statistical approach have been described to assess the functional connectivity by rest-fMRI. There are mainly three widely used techniques for rests-fMRI connectivity analysis: the seed-based analysis, the independent component analysis (ICA), and the graph theory analysis. The seed-based analysis was the first method used to identify the resting state networks by the selection of a seed region of interest (ROI) to locate by computational and statistical analysis the linear correlation of the seed regions with all the other voxels of the entire brain [18,40,41]. The ICA method investigates multiple simultaneous voxels to voxel interactions of distinct networks in the brain, where the low-frequency spontaneous fluctuations of the rest-fMRI signal may be automatically recovered from the noise [42,43,44].

**Figure 5:**
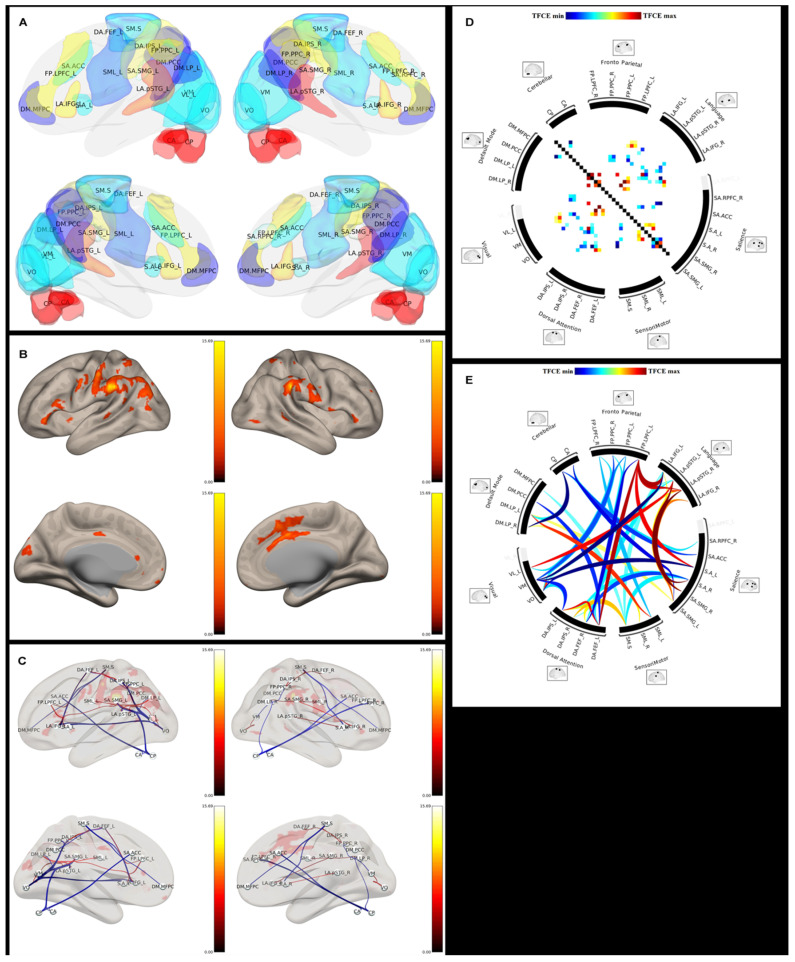
The graph theory analysis is used to define a mathematical model of complex network functions within the human brain and it allows to represents the (**A**) resting-state networks based on (**B**) BOLD signal fluctuation by (**C**) groups of nodes connected by edges. (**D**) Circular matrix defines the mathematical model of network connection and the (**E**) circular graph connectome represents the functional connections between networks. The thickness of the line connecting the RSNs around the edge of the circular connectome indicates the strength of the connection.

The graph theory analysis is used to define a mathematical model of complex network functions within the human brain. With this method, the resting-state networks are represented by groups of nodes connected by edges [5,6,45,46] (Figure 5).

Therefore, the graph theory analysis shows the relation between the nodes and edges and describes these interactions through different graph parameters (e.g., clustering coefficient). Using these analysis methods for functional connectivity, different RSNs in the brain can be identified, such as the salience network, auditory network, basal ganglia network, higher visual network, visuospatial network, default mode network, language network, executive network, precuneus network, primary visual network, and sensory-motor network. Importantly, for rest-fMRI image processing, a variety of different post-processing software packages can be used, mainly freely available as open-sources, such as Statistical Parametric Mapping (SPM Available online: https://www.fil.ion.ucl.ac.uk/spm/ (accessed on 1 September 2021)), FSL (Available online: https://fsl.fmrib.ox.ac.uk/fsl/fslwiki (accessed on 1 September 2021)), Analysis of Functional NeuroImages (AFNI Available online: https://afni.nimh.nih.gov/ (accessed on 1 September 2021)), and CONN toolbox (Available online: https://web.conn-toolbox.org/ (accessed on 1 September 2021)). However, these software require a high knowledge and expertise to conduct postprocessing analysis as they lack standardization, thus requiring ad hoc adjustments or custom-made scripts to tailor the postprocessing pipeline.

Single-subject and group rest-fMRI analysis for the commonly used functional networks, can be performed and graphically displayed as a circular graph named “connectome” (Figure 2) [5,47].

**Figure 6:**
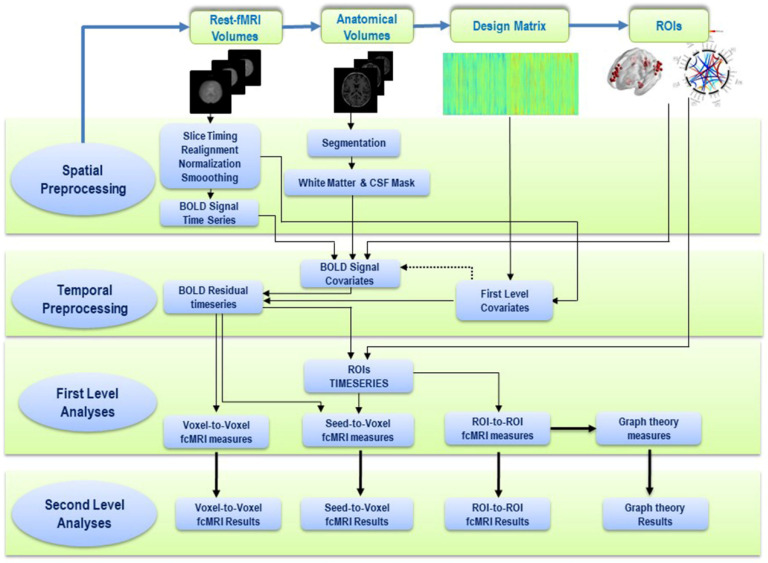
Rest-fMRI workflow pipeline for data extraction from MR images and data analysis using graph theory.

**Figure 7:**
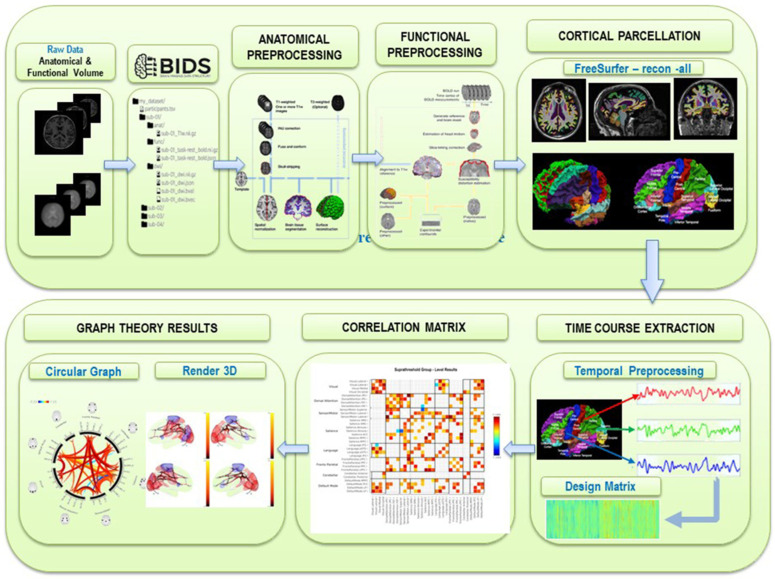
Rest-fMRI custom workflow for brain MR images anatomical preprocessing, functional preprocessing, cortical parcellation, and time course extraction used for correlation matrix and to compute graph theory results.

An overview of the workflow routinely used in our institution, consisting of rest-fMRI post-processing and analysis pipeline is shown in Figure 6 and Figure 7. Real-time quality control using the processing pipeline is performed during real-time rest-fMRI acquisition. A trained observer evaluates the quality control parameters on the MR acquisition workstation to determine the scan success or repeat the scan after instructing the patient to reduce their head motion or to improve their compliance. Offline quality control and correction of rest-fMRI images is done, and analysis of connectome is assessed by co-registration with structural MRI to measure the Euclidean distances between intraoperative mapping coordinates and the edges of the corresponding connectivity and activation clusters using the graph theory analysis. The entire preprocessing and post-processing pipeline can be implemented using various procedures and opensource fMRI analysis software.

In our clinical setting, we implemented a post-processing pipeline based on the opensource software fMRIPrep (Available online: https://fmriprep.org/en/stable/ (accessed on 1 September 2021)) and FreeSurfer (Available online: https://surfer.nmr.mgh.harvard.edu/ (accessed on 1 September 2021)), and the Conn Toolbox (Available online: https://web.conn-toolbox.org/ (accessed on 1 September 2021)) running under MatLab (MathWoks, Natick, MA, USA, Available online: https://it.mathworks.com/products/matlab.html (accessed on 1 September 2021)), using a 64-bit Intel-based High Performance Computer (HPC) with a 16 core CPU, 64 GB RAM, nVidia Tesla 100 graphic board, and 7 TB storage hard disk.

**Figure 8:**
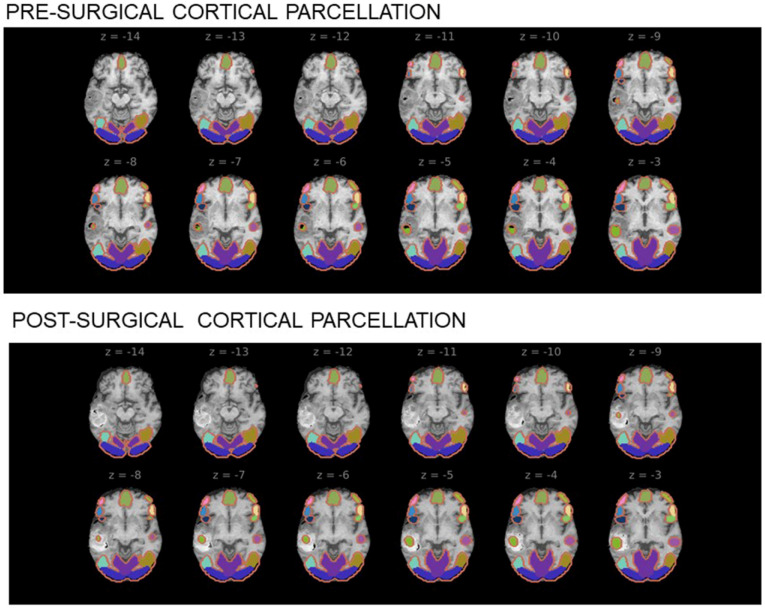
Cortical parcellation obtained before and after surgical resection of brain glioma.

The preprocessing steps of the rest-fMRI steps included: rigid body motion correction, spatial smoothing (4 mm Gaussian spatial filter kernel), spatial normalization using the Montreal Neurological Institute (MNI) atlas (Available online: https://brainmap.org/training/BrettTransform.html (accessed on 1 September 2021)) on the subjects, and a low pass filter to reduce signal fluctuations due to cardiac and respiratory pulsations. A 10% intensity threshold was applied to the raw images to remove spurious correlations outside of the brain. Atlas-based cortical parcellation before and after surgery was obtained with FreeSurfer (Figure 8), and seed selection was carried out using Brodmann areas (BAs) (Available online: https://www.brainm.com/software/pubs/dg/BA_10-20_ROI_Talairach/functions.htm (accessed on 1 September 2021)) after transforming the coordinates from the MNI atlas into the Talairach atlas (Available online: https://brainmap.org/training/BrettTransform.html (accessed on 1 September 2021)). The sensorimotor network (SMN), encompassing primary sensory and motor areas, was mapped using the atlas-based left BAs 1–3 (BA1-3L). The language network was mapped using either left Bas 44 and 45 (BA44, 45L) for Broca’s area or left BAs 22, 39, and 40 (BA22,39,40L) for Wernicke’s area. Detailed post-processing steps used included each 3D MPRAGE T1w (T1-weighted) volume being corrected for intensity non-uniformity and skull-stripped. Brain surfaces were reconstructed with recon-all of FreeSurfer, and the brain mask estimated previously was refined with FreeSurfer-derived segmentation of the cortical gray-matter. Spatial normalization was performed through nonlinear registration using brain-extracted versions of both T1w volume and template. Brain tissue segmentation of cerebrospinal fluid (CSF), white-matter (WM) and gray-matter (GM) was performed on the brain-extracted 3D MPRAGE T1w with FSL (Available online: https://fsl.fmrib.ox.ac.uk/fsl/fslwiki/FSL (accessed on on 1 September 2021)). EPI rest-fMRI images were slice time-corrected using AFNI (Available online: https://afni.nimh.nih.gov/ (accessed on 1 September 2021)) and motion-corrected using FSL. This was followed by co-registration to the corresponding 3D MPRAGE T1w images using boundary-based registration with six degrees of freedom, using FreeSurfer. Motion-correcting transformations, BOLD-to-T1w transformation, and T1w-to-template (MNI) warp were concatenated and applied in a single step using ANTs (Avatailable online: https://github.com/ANTsX/ANTs (accessed on 1 September 2021)).

Physiological noise regressors were extracted applying CompCor [47]. Principal components were estimated for the two CompCor variants: temporal (tCompCor) and anatomical (aCompCor). A mask to exclude signal with cortical origin was obtained by eroding the brain mask, ensuring it only contained subcortical structures. Six tCompCor components were then calculated including only the top 5% variable voxels within that subcortical mask. For aCompCor, six components were calculated within the intersection of the subcortical mask and the union of CSF and WM masks calculated in T1w space, after their projection to the native space of each functional run. ICA-based Automatic Removal of Motion Artifacts (AROMA) [48] was used to generate aggressive noise regressors as well as to create a variant of data that is non-aggressively denoised.

This method provides the surgeon information about the exact localization of resting-state networks owing to the decision of the best surgical approach for the extent of resection (EOR). Indeed, functional network preservation should be considered when evaluating the EOR planning. In this setting, rest-fMRI can predict the impairment in the executive functions or other cognitive processes after surgical resection of a brain tumor, and can be valuable in identifying behavioral changes.

Rest-fMRI can thus contribute to tailoring a personalized surgery for brain tumor resection improving the surgeon’s ability to increase the EOR, and simultaneously minimize the risk of impairment of critical functional networks responsible for specialized function [5].

Rest-fMRI can also play a role in the postoperative evaluation of patients after tumor resection by estimate the potential alterations in functional connectivity and the onset of new neurologic deficits due to surgical resection. Moreover, in longitudinal studies, rest-fMRI allows assessing functional reorganization after surgical resection of brain tumors. Therefore, this advanced neuroimaging tool may be implemented in the routine clinical management of brain tumor patients.

Despite further validation is currently necessary in a large population of patients to define the clinical value of task-driven fMRI and rest-fMRI for neurosurgical planning of brain tumors [48,49,50,51,52,53,54,55], in our experience, the decision making process for brain tumor surgical planning was improved with the use of rest-fMRI as it was valuable in the assessment of feasibility of resection, surgical planning, and selection of patients for invasive functional mapping procedures, as reported in other studies [56]. Rest-fMRI was helpful in 87% of patients with sensorimotor eloquent lesions, and in 85% of patients with language eloquent lesions. Moreover, preoperative rest-fMRI has influenced the decision-making in brain tumor resection by adjustment of the treatment plans, resulting in reduced surgical time, increased extent of resection, and decreased craniotomy size, as reported in previous studies using task-driven fMRI [57].

## 5. Limitations

There are still various problems affecting the performance and analysis of rest-fMRI in the clinical setting of brain tumors. The susceptibility artifacts and neurovascular uncoupling (NVU) issues, as well as head motion artifacts, which result in rest-fMRI image quality degradation. Susceptibility effects are more evident at 3T and higher field strength. They reflect the extent of magnetization of a substance in the presence of an external magnetic field. Field distortions and MR artifacts are commonly present around metal objects and implants, as they contain ferromagnetic materials which is perturbative to the external magnetic field. Susceptibility-induced signal loss from T2*-dephasing results in “geometric distortion” affecting rest-fMRI acquisition which is worse at high field strength [5,6].

Head motion and physiologic disturbance due to respiratory and cardiac cycles are sources of artifacts that heavily affect rest-fMRI. Head restraints used to reduce the effects of head motion and compensation of respiratory fluctuations and cardiac pulsations can achieve an improvement of these sources of artifacts. False-positive BOLD activation due to eye motion, random noise, partial volume effects, and physiological pulsations, along with post-processing statistical issues, may reduce the reliability of rest-fMRI studies [5,6,58].

To address these issues, retrospective motion and physiological noise correction methods can be used. Correction for the non-neural noise from white matter and cerebrospinal fluid (known as “nuisance regression”) can be utilized to improve the image quality of the rest-fMRI dataset. [5,6,58]. Impaired blood oxygenation level-dependent (BOLD) fMRI activation in the eloquent cortex in the vicinity of brain tumors can be a source of inaccurate pre-surgical planning evaluation that can lead to inadvertent eloquent cortical resection. These abnormal BOLD activations close to focal brain tumors often occur due to the disruption of coupling between neuronal activity and adjacent microvasculature, known as “neurovascular uncoupling” (NVU) [58].

Another limitation to this approach is the heterogeneity of lesion localization and spatial extent is altered anatomy due to a lesion mass effect that impacts cortical parcellation and seed selection [51]. Automation of spatial normalization using improved co-registration, spatial normalization, and tissue segmentation is widely available in opensource software packages, such as SPM, FreeSurfer, and is expected to improve workflow.

Artificial intelligence-based classification of resting-state connectivity is expected to provide more precise analysis results. This may ultimately allow integration of real-time connectivity maps into neurosurgical guidance.

## 6. Conclusions

Rest-fMRI is an advanced neuroimaging technique able to provide useful information concerning different neuronal networks and their alterations related to the tumor and surgery. It provides an assessment for diagnosis, prognosis, and personalized treatment, which can be improved with the complementary information obtained by diffusion tractography [59] and standard morphologic magnetic resonance imaging. Moreover, rest-fMRI can be the unique method of assessing functional connectivity for surgical planning of brain tumors when a patient cannot perform task-driven-fMRI.

This approach has the potential for real-time pre-surgical mapping of eloquent cortex in patients with brain tumors. Future developments include parcellation of eloquent cortex based on combining rest-fMRI with diffusion tensor imaging (DTI) [60] by means of machine learning artificial intelligence to assess the integrity of fiber tracts in the vicinity of a tumor, and MRI spectroscopy to delineate tumor margins and identify infiltration.

Standardization and validation of advanced imaging techniques and post-processing of fMRI will improve the risk–benefit assessment for each patient allowing neurosurgeons to better understand the clinical outcome of resection.

## Figures and Tables

**Table 1:** Brain resting-state networks (RSNs) nomenclature.

Id	Extensive Name	Short Name	Id	Extensive Name	Short Name
1	DefaultMode.MPFC	DM.MFPC	17	Salience.SMG l	SA.SMG_L
2	DefaultMode.LP l	DM.LP_L	18	Salience.SMG r	SA.SMG_R
3	DefaultMode.LP r	DM.LP_R	19	DorsalAttention.FEF l	DA.FEF_L
4	DefaultMode.PCC	DM.PCC	20	DorsalAttention.FEF r	DA.FEF_R
5	SensoriMotor.Lateral l	SML_L	21	DorsalAttention.IPS l	DA.IPS_L
6	SensoriMotor.Lateral r	SML_R	22	DorsalAttention.IPS r	DA.IPS_R
7	SensoriMotor.Superior	SM.S	23	FrontoParietal.LPFC l	FP.LPFC_L
8	Visual.Medial	VM	24	FrontoParietal.PPC l	FP.PPC_L
9	Visual.Occipital	VO	25	FrontoParietal.LPFC r	FP.LPFC_R
10	Visual.Lateral l	VL_L	26	FrontoParietal.PPC r	FP.PPC_R
11	Visual.Lateral r	VL_R	27	Language.IFG l	LA.IFG_L
12	Salience.ACC	SA.ACC	28	Language.IFG r	LA.IFG_R
13	Salience.AInsula l	S.A_L	29	Language.pSTG l	LA.pSTG_L
14	Salience.AInsula r	S.A_R	30	Language.pSTG r	LA.pSTG_R
15	Salience.RPFC l	SA.RPFC_L	31	Cerebellar.Anterior	CA
16	Salience.RPFC r	SA.RPFC_R	32	Cerebellar.Posterior	CP

## Data Availability

Proper links are provided in the manuscripts therefore this statement can be excluded.
